# Hypoxia-inducible tumour-specific promoters as a dual-targeting transcriptional regulation system for cancer gene therapy

**DOI:** 10.3332/ecancer.2017.751

**Published:** 2017-07-06

**Authors:** Bita Javan, Majid Shahbazi

**Affiliations:** Medical Cellular and Molecular Research Center, Golestan University of Medical Sciences, Gorgan 4934174515, Iran

**Keywords:** : hypoxia, hypoxia-responsive element (HRE), transcriptional targeting

## Abstract

Transcriptional targeting is the best approach for specific gene therapy. Hypoxia is a common feature of the tumour microenvironment. Therefore, targeting gene expression in hypoxic cells by placing transgene under the control of a hypoxia-responsive promoter can be a good strategy for cancer-specific gene therapy. The hypoxia-inducible gene expression system has been investigated more in suicide gene therapy and it can also be of great help in knocking down cancer gene therapy with siRNAs. However, this system needs to be optimised to have maximum efficacy with minimum side effects in normal tissues. The combination of tissue-/tumour-specific promoters with HRE core sequences has been found to enhance the specificity and efficacy of this system. In this review, hypoxia-inducible gene expression system as well as gene therapy strategies targeting tumour hypoxia will be discussed. This review will also focus on hypoxia-inducible tumour-specific promoters as a dual-targeting transcriptional regulation systems developed for cancer-specific gene therapy.

## Introduction

Hypoxia, indicating solid tumours, may prove to be an ideal target for tumour-specific gene therapy. Hypoxia-inducible factors (HIFs), HIF-1 and HIF-2, chiefly mediate the hypoxic response. Numerous target genes (e.g., *VEGF-A*, *Epo*) containing regulatory elements in their promoter regions, known as hypoxia-response elements (HREs) that serve as the binding site of HIFs, have been identified [1, 2]. Several studies have used HRE sequences in combination with a minimal promoter to restrict the gene expression in the hypoxic tumour environment [3–7]. A hypoxia-inducible gene expression system has been investigated majorly in the suicide gene therapy studies to propel drive selective prodrug activating enzyme expression under hypoxic conditions [8, 9]. Moreover, these studies have also considered hypoxia-mediated proapoptotic gene expression [10, 11].

The hypoxia-induced system has certain limitations. This system is leaky and reveals basal promoter activity under normoxia, which may lead to side effects. Therefore, tight regulation of gene expression is necessary to minimise the adverse effects on normal tissues. One possible strategy is to combine the HRE sequences to the tissue-specific promoter in order to induce a high level of specificity in the cancer gene therapy. This dual-specific expression system has been previously applied in cancer gene therapy [10, 12, 13]. In addition, this system in combination with other approaches, including radiotherapy and chemotherapy, would be effective in sensitising the resistant tumours to these treatments.

This review discusses hypoxia and mechanisms for hypoxia-inducible gene expression, as well as gene therapy strategies targeting tumour hypoxia. Moreover, it also emphasises on several tissue and tumour-specific promoters, along with their application in cancer gene therapy. Eventually, it describes the dual-targeting transcriptional regulation systems with an emphasis on hypoxia-inducible tumour-specific promoters for hypoxia and tissue-specific gene therapy.

## Tumour hypoxia and hypoxia-inducible factors (HIFs)

Hypoxia is a key feature of solid tumours [14]. The rapid growth of tumour cells and their inability to form normal blood vessels reduce the blood supply and oxygen tension. Therefore, the oxygen concentration in the tumour microenvironment drops to hypoxic levels below 2% [15, 16]. Under these conditions, the hypoxic tumour cells that maintain their ability of growth and proliferation become more resistant to chemotherapy and radiotherapy, and these cells exhibit aggressive and metastatic behaviour [17–19].

**Figure 1.**
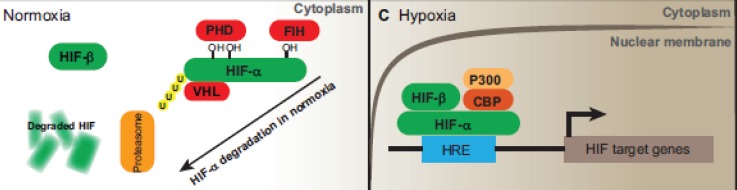
Schematic diagram of the HIF-signalling system. Under normal oxygen conditions, the HIF-1α protein becomes hydroxylated by prolyl hydroxylase (PHD) and ubiquinated, in which case it will be degraded by proteasomes. In the setting of hypoxia, it binds to ubiquitously expressing HIF-1β to form a heterodimer. The heterodimer then translocates to the nucleus and binds to HRE elements in the promoter/enhancer region of target genes, inducing the expression of various HIF-1-responsive genes. Adapted from [65].

HIFs are essential for cellular hypoxia adaptation during low oxygen levels and regulate the expression of various genes involved in the glucose homeostasis, cell proliferation, apoptosis, differentiation, angiogenesis, vascular permeability and inflammation. HIFs are heterodimeric proteins comprising hypoxia-inducible α subunit, with three isoforms: HIF-1a, HIF-2a and HIF-3a; and a constitutively expressed nuclear β subunit (also known as ARNT, the aryl hydrocarbon receptor nuclear translocator), with only two isoforms: HIF-1b and HIF-2b [2, 20]. Under normoxia, α subunit is inactive and is located in a proteasome degradation pathway; however, under hypoxia, it is stabilised and accumulates in the cells, following which it transferred to the nucleolus where it forms a dimer with the β subunit. Eventually, this α–β active complex binds to the hypoxia-response element (HRE) with the consensus core sequence 5′-(A/G)CGT(G/C)(G/C)-3′ in the target genes (Figure 1) [21, 22]. HREs are enhancers located at varying positions and orientations of the coding regions of oxygen-responsive genes [22–24].

## Hypoxia as a target for tissue-specific gene therapy in cancer

Several gene therapy methods reveal certain limitations including the specific targeting of the transgene to tumour tissues, especially solid tumours. To solve this problem, various strategies have been developed. Of these approaches, one strategy that is discussed in this review is to apply hypoxia-inducible gene expression systems. Solid tumours, characterised by the presence of hypoxic regions, constitute around 90% of cancers and are a suitable target for hypoxia-inducible gene therapy [23].

Hypoxia-targeting methods may prove to be effective in selective cancer gene therapy and also in the current treatment strategies by reducing the tumour tissue resistance to radiation therapies and chemotherapeutic drugs. Application of hypoxia-inducible gene expression systems is not limited to cancer treatment and has been used in treating other ischemic diseases in which hypoxia is a common feature, such as ischemic heart disease and ischaemic stroke [25, 26].

In general, hypoxic tumours targeting gene therapy strategies are classified into the following categories: transcriptional regulation with a hypoxia-inducible promoter, which is the most widely used strategy and is also addressed in this article; post-transcriptional and post-translational regulation for higher stability of mRNA; and protein under hypoxia [27–29].

## Hypoxia-inducible gene expression systems

**Figure 2.**
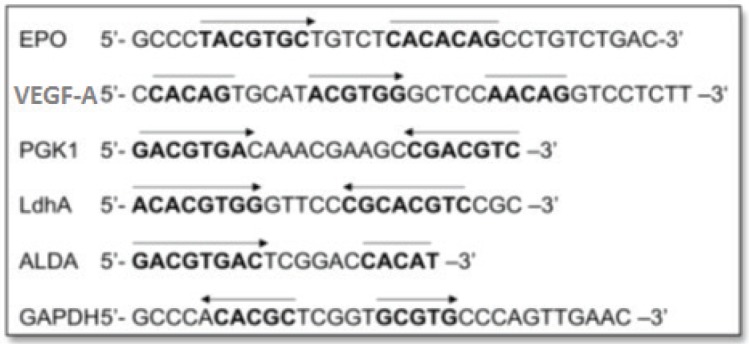
HREs from the Epo, VEGF-A, PGK1, Ldha, ALDA and GAPDH gene. Functional HIF-1-binding sites (arrow) and sequences with > 4/5 match to the functionally essential Epo sequence 5′CACAG-3′ (overline). Adapted from [24] and [34].

Hypoxia-inducible gene expression systems have been constructed from the HRE sequence binding to a basal promoter. The HRE sequences have been isolated from various HIF-1-responsive genes (listed in Figure 2), among which the most commonly exploited genes for selective gene therapy in the HIF-1-active microenvironment are erythropoietin (*Epo*), phosphoglycerate kinase 1 (*PGK1*), vascular endothelial growth factor A (*VEGF-A*) and lactate dehydrogenase A (*LDH A*) [1, 29–34].

Several constructs of HRE sequences and a minimum viral promoter such as SV40, cytomegalovirus (CMV) and adenovirus E1B have been developed, which are chiefly investigated in gene-directed enzyme prodrug therapy (GDEPT) approaches. In addition, few studies reported that HIF-1-dependent induction of enzymes used in GDEPT such as the herpes simplex virus thymidine kinase (HSV-TK) and a bacterial cytosine deaminase, followed by treatment with their related prodrugs, resulted in the suppression of tumour growth and/or enhanced radiotherapy effects. Some of these studies are further described in this section [9, 35].

## Hypoxia-inducible gene expression systems in cancer gene therapy

Target specificity and therapeutic effects of gene therapy using hypoxia-inducible gene expression systems have been assessed in various cancers *in vitro* and *in vivo*. Dachs *et al* initially constructed the hypoxia-inducible system for gene therapy by applying HRE from the PGK-1 promoter in combination with the SV40 promoter for the expression of the suicide gene deoxycytidylate deaminase (CD) to sensitise the hypoxic tumour cells to the prodrug 5-fluorocytosine (5FC) [36]. Tumour cells in acute hypoxia region remain viable and are more resistant to chemotherapy and radiotherapy; hence, a suitable approach would be to target these areas within the tumour tissues by hypoxia-inducible vectors. For this purpose, PGK-1 HRE was applied to HSV-TK expression in response to acute hypoxia in the stroma. Ingram and Porter generated retroviral vector by incorporating HREs from the *PGK-1* gene within long terminal repeats to target the transient hypoxia within the vasculature and stroma [37].

Similarly, VEGF-A HRE–HSV-TK were applied in renal cell carcinoma (RCC) suicide gene therapy [38]. This system can target both hypoxic cells and RCC with *VHL* mutations because *VHL* gene contributing to the destruction of HIF-1 proteins in normoxia is inactive in 75% of RCC. Retroviral vector harbouring the HSV-TK under the control of the *VEGF* promoter, LV(HRE)TK, was used in suicide gene therapy in Lewis lung carcinoma [39]. A11 cells transfected with LV(HRE)TK were more sensitive to ganciclovir (GCV) than the cells transfected with the control vector (harbouring VEGF promoter alone), and the sensitivity of these cells was increased by exposure to hypoxia. Moreover, LV(HRE)TK vector was also effective in suppressing the tumour growth *in vivo*. This approach is more efficient in targeting and treating highly metastatic tumour cells that overexpress VEGF. HREs from *GAPDH* and *Epo* were also evaluated for cancer-specific gene therapy [40, 41].

Recently, hypoxia-inducible TK/GCV+CDUPRT/5-FC triple suicide gene therapy has been reported to specifically target and sensitise the hypoxic tumour cells for radiation both *in vitro* and *in vivo* with GCV and 5-FC treatments [42]. In this study, HCT-8 cells were cotransfected with two hypoxia-inducible expression vectors; p9HRE-TK/eGFP containing nine tandem repeats of human *EPO* gene HRE linked to the SV40 minimal promoter (SV40 min) and p9HRE-CD/UPRT/mDsRed, which contains the CD, uracil phosphoribosyltransferase (UPRT), and mDsRed fusion gene under the regulation of the 9HRE/SV40 min. Radiosensitisation effect is known to be a potential advantage of suicide gene therapy. Previous studies have suggested that radiosensitisation in HSV-1 TK/GCV and CD/UPRT/5-FC approaches is mediated through the inhibition of the repair of radiation-induced sublethal DNA damage and thymidylate synthase, respectively, resulting in an increased DNA strand break. Another advantage of this approach is the bystander effect. Hypoxia-inducible TK/GCV+CDUPRT/5-FC triple suicide gene therapy leads to greater cytotoxicity than any of these approaches when used alone, which demonstrated the superior bystander killing effect of this method.

In another study, hypoxia-responsive promoter and repeated targeting sequences of a miRNA were used to construct the neural stem cells (NSC) vector with high targeting specificity for cancer gene therapy *in vitro* and in mouse tumour model [43]. This combinatorial NSC vector comprises an artificially optimised promoter optHRP (composed of four optimised HREs followed by a TATA box) and miR-199a-5p-targeting sequences. MiR-199a-5p is an inhibitor of HIF-1α and its expression level decreases in response to hypoxia. Therefore, it can potentially block the transgene expression in normoxia.

In addition to the GDEPT approach, another possible therapeutic application of HIF-1-dpendent promoters is the expression of cytotoxic genes such as proapoptotic genes (e.g., BAX and harakiki) under the hypoxic condition [10, 11, 44].

## Optimisation of HIF-1/HRE system

Several factors affect the activity of synthetic hypoxia-responsive promoters; these include the following:

- Nature of the minimal promoter: The optimal expression of the transgene under hypoxia depends on the selected promoter sequence. For instance, replacing the minimal thymidine kinase (TK) promoter with the minimal SV40 promoter results in an increased induction ratio of 18-fold to 146-fold [35, 45].
- HRE copy number: Increasing the HRE copy number is a preferable option to promote enhanced gene expression [4–6, 36, 46, 47]. Multimerisation of the HRE core sequence in different gene, such as *Epo/VEGF-A*, results in increase in the basal gene expression under hypoxia when compared to the aerobic controls, with a threshold of a minimum three copies. The effect of HRE copy number on gene expression was under debate. For constructs containing more than 5–8 copies, several studies reported possible saturation effect of HRE copy numbers.
- The origin of HRE sequences: HREs isolated from various HIF-1-responsive genes reported different induction levels of gene expression; among these, HRE from VEGF-A gene reveals the highest inducibility in hypoxia and lower baseline activity in normoxia [4, 5, 33, 45–49].
- Oxygen tension: The oxygen levels vary throughout the tumour and ranges from 2 mmHg (0.3% O2) to 18 mm Hg (2.4% O_2_). It is important for HRE promoter to have inducible activity at O_2_ levels of ~1% which depends on its copy number [5, 48, 50].
- Gene profile of tumour cells: Since oncogenes and tumour suppressor genes may affect the HIF subunits expression, the tumour cells may exhibit distinct responses to hypoxia based on their gene profile. For example, tumour cells with a wild type p53 gene may have lower hypoxia-induced gene expression than those, which bear the mutant type [51, 52].

## Tissue-/tumour-specific promoters for cancer gene therapy

Viral promoters have strong promoter activity and are optimal during high levels of transient expression required for a cytotoxic or immunostimulatory effect; however, they have certain limitations, including lack of specificity, immunogenicity, as well as a short-term expression *in vivo*.

Several tumour-specific promoters, such as survivin and carcinoembryonic antigen (CEA), were evaluated for specific cancer gene therapy to restrict the expression of the transgene in tumour tissues and to reduce the undesired side effects in normal tissues [53, 54]. In addition, many tissue-specific promoters, such as prostate-specific membrane antigen (PSA/PMSA), have also been applied to cancer and other genetic disorders including liver disorders and vascular diseases [55]; however, the utility of these promoters is limited due to their relative weakness and natural activity in normal tissues. Several strategies are available to overcome these problems. Tissue-specific promoters can be combined with the transactivators such as the two-step transcription amplification systems, the most widely used transactivator system, to facilitate transgene expression. The other strategies include the use of cell-cycle elements, tissue differentiation factors, hormones, cytokines, chemicals or physical stimuli. For example, a tumour-specific promoter can combine with a regulatory element such as an HRE sequence to transcriptionally target the hypoxic tumours [56, 57].

## Hypoxia-inducible tissue-specific promoter

Hypoxia-inducible gene expression system is not an on/off system. Hypoxia-inducible promoters have basal promoter activity under normoxia, which may lead to side effects. Moreover, this system is not suitable for systemic delivery due to varying amount of oxygen tensions in the most of human tissues that ranges 2–9% [58]. Therefore, for safe cancer gene therapy, tight regulation of gene expression is required to avoid the side effects in normal tissues.

Two important strategies may be used to improve the specificity and efficiency of the hypoxia-regulated system. Since HIF-1/HRE system has an advantage of combining with various promoters, one possible strategy is the combination of HRE sequence to the tissue specific promoter to induce a high level of specificity in the cancer gene therapy (Table 1). A combination of oestrogen response elements (ERE) and HRE from PGK-2 is an example of this system, which has been used for transcriptional targeting of breast cancer gene therapy [10]. A novel conditionally replicative adenovirus vector was constructed by placing two viral transcription units, E_1_a and E_4_, which were controlled by an artificial hybrid promoter (ERE/HRE promoter), preferentially active in the ER+ breast cancer cells or cells growing under hypoxic conditions. To confirm the therapeutic effect of a hybrid promoter, MCF-7 cells were transfected with an expression vector containing proapoptotic harakiri gene under the control of the ERE/HRE promoter. Transfected cells became apoptotic in the presence of oestrogens, while this effect was partially blocked by the antioestrogen drug. Additionally, the hypoxia treatment also activated the apoptotic effect of the expression vector. Another dual-specificity promoter includes the combination of survivin promoter (Sur-P) and HRE from the VEGF-A promoter [12]. HRE/Sur-P induces the expression of the caspase-3 gene under hypoxia, which resulted in apoptosis in breast cancer cells. In another study, survivin and MUC1 promoters have been combined with HRE and ERE to construct HRE–ERE-MUC1 promoter and HRE–ERE-Sur promoter, respectively [59]. These dual-specificity promoters provide higher and more specific level of tBid (truncated Bid) expression in breast cancer cells.

In addition to breast cancer, this dual-targeting transcriptional system has been applied in other cancer gene therapies. For example, hypoxia/hepatoma dual-specific suicide gene expression vector has been exploited for hepatocellular carcinoma-specific gene therapy [13]. This two-step transcription amplification system comprised two regulatory elements, Epo HRE and AFPL promoter (alpha-fetoprotein (AFP) promoter with enhancer regulatory region (~0.4 kbp), which has a higher hepatoma specificity than the AFP promoter alone). To confirm the efficacy of this vector in cancer gene therapy, the hypoxia/hepatoma dual-specific pEpo-AFPL-TK vector was constructed, which showed higher apoptosis under hypoxia in the hepatoma cells than the control and pSV-HSV-TK groups. Another example is the hypoxia and glioma dual-specific HS-TK gene expression system, pEpo-NI2-SV-HSV-TK, comprising the Epo enhancer and nestin intron 2 (NI2), which developed the glioblastoma-specific suicide gene expression both *in vitro* and *in vivo* [60]. This dual-targeting system reported increased specificity of the HSV-TK expression in the ischaemic glioblastoma tissues and reduced the non-specific expression in other tissues.

Hypoxia/radiation dual sensitive chimeric HRE/early growth response 1 (Egr 1) promoter is a dual-targeting vector, which induces expression of the proapoptotic second mitochondria derived activator of caspases (Smac) gene in lung adenocarcinoma cells subjected to hypoxia and X ray irradiation [61]. Another example this system application in gene radiotherapy is the combination of Epo/E9 enhancer; five copies of HRE and nine copies of radiation response element (CArG); and whey acidic protein (WAP) promoter, a mammary-specific murine promoter, producing the radiation induced over expression of a transduced reporter gene under hypoxia both *in vitro* and *in vivo* [62].

Several studies in this field emphasise on applying hypoxia-inducible gene expression system in suicide gene therapy. Considering the significance of tight regulation of shRNA expression to avoid potential side effects, these systems may also be useful in knocking down cancer gene therapy with siRNAs.

Nevertheless, issues that still remain unsolved include poor activity of tissue-specific promoters, their leaky expression in normal tissues as well as gene, and drug delivery to hypoxic tumour regions. Certain strategies involving the transactivator and posttranscriptional regulation (e.g., using an untranslated region) can increase the gene expression in hypoxic tumour tissue. Moreover, the addition of oxygen-dependent degradation (ODD) domain as a post-translational regulation may be effective in reducing the side effects in normal tissue [29, 30, 63, 64].

## Conclusions

Hypoxic tumour regions are exceptional targets for cancer-specific gene therapy. These regions are targeted by a combination of HREs sequences of hypoxia-responsive genes with a core promoter to limit the therapeutic gene expression in tumour tissues. Studies have demonstrated the potential of hypoxia-inducible gene expression systems for tumour-selective gene therapy. Furthermore, this system can be a good complement to the current cancer treatments. For example, this system can be applied in gene radiotherapy using a combination of radiation response element with HRE sequences as a hypoxia- and radiation-inducible promoter to sensitise the resistant tumour cells to radiation [61, 62].

Previous studies have determined that the optimal expression of HIF-1/HRE gene expression system depends on the selected core promoter sequence [37, 49]. In addition, spacing and rearrangement of the HRE sequence and its copy numbers are additional factors that may impact the efficacy of this gene expression system [4–6, 46, 47]. In primary studies, viral core promoters were used by HRE sequences to induce hypoxia gene expression that was problematic due to immunogenicity, lack of specificity and short-term expression *in vivo*.

Moreover, hypoxia-inducible system, like other inducible systems, has leaky expression causing an undesired effect on the normal tissues. To overcome these limitations, tissue-/tumour-specific promoters were combined with HREs to construct a dual-specificity gene expression system to increase the specificity and minimise the side effects; however, these promoters are not as strong as viral promoters and often lack sufficient specificity. Therefore, extra regulatory elements such as transactivators, in combination with tissue-/tumour-specific promoters, can be used to increase their expression activity. Post-transcriptional and post-translational strategies have also been applied to provide tight regulation and to limit the gene expression in tumour cells. Additionally, translocational targeting can provide high targeting specificity and consequently reduced nonspecific effect [29, 30, 63, 64].

Due to various responses to hypoxia exposure in tumour cells, evaluating the efficacy of the hypoxia-inducible system *in vivo* and in different tumours is essential for the development of hypoxia-regulated cancer gene therapies. Nevertheless, several studies in this field have emphasised on the application of this system in breast cancer gene therapy.

Consequently, optimisation of such dual targeting system and expanding their applications, in combination with other approaches such as radiotherapy, would be a promising approach to develop strategies with high targeting specificity for cancer gene therapy.

## Figures and Tables

**Table 1.** Dual-targeting gene expression systems for cancer gene therapy.

Reference	Cancer	Target gene	Regulatory elements
R Hernandez-Alcoceba *et al* (2001) [10]	Breast cancer	Harakiri	ERE-+HRE from the PGK-1 gene
L Yang, Z Cao *et al* (2004) [12]	Breast cancer	Caspase-3	Survivin promoter+HRE from the VEGF-A gene
Lipnik K *et al* (2006) [62]	Breast cancer	EGFP	WAP promoter+ CArG+HRE from the Epo gene
Farokhimanesh S *et al* (2010) [59]	Breast cancer	tBid	MUC1 / Survivin promoter+ERE+ HRE
Kim HA *et al* (2013) [13]	Hepatocellular carcinoma	TK/ (GCV) prodrug	AFP promoter + HRE from the Epo gene
Kim HA *et al* (2014) [60]	Glioblastoma	TK/ (GCV) prodrug	Nestin promoter and NI2 + HRE from the Epo gene
Li CF *et al* (2014) [61]	Lung adenocarcinoma	Smac	Early growth response 1 (Egr 1) promoter + HRE
